# Biomechanical Evaluation of CAD/CAM Inlay Restorations Through Experimental Flexural Strength Testing and Finite Element Analysis

**DOI:** 10.3390/jfb17030123

**Published:** 2026-03-03

**Authors:** Omer Sagsoz, Mehmet Yildiz, Hojjat Ghahramanzadeh Asl

**Affiliations:** 1 Department of Restorative Treatment, Faculty of Dentistry, Atatürk University, 25240 Erzurum, Türkiye; omer.sagsoz@atauni.edu.tr; 2 Department of Restorative Treatment, Faculty of Dentistry, Sakarya University, 54100 Sakarya, Türkiye; 3 Department of Mechanical Engineering, Karadeniz Technical University, 61080 Trabzon, Türkiye; h.kahramanzade@ktu.edu.tr

**Keywords:** CAD/CAM materials, finite element analysis (FEA), flexural strength, inlay restoration, modulus of elasticity, nanoindentation, von Mises stress

## Abstract

Background: This study aimed to investigate the biomechanical behavior of conservative inlay restorations fabricated from different CAD/CAM materials by combining experimental flexural strength testing with finite element analysis. Methods: Five CAD/CAM materials were evaluated: feldspathic ceramic (Cerec Blocs), leucite-reinforced ceramic (IPS Empress CAD), resin nano-ceramic (Lava Ultimate), polymer-infiltrated ceramic network (VITA Enamic), and lithium disilicate ceramic (IPS e.max CAD). Young’s modulus and Poisson’s ratio were experimentally determined using three-point bending and nanoindentation tests and used as inputs for 3D FEA. Von Mises (VM) stress distributions within the inlays were analyzed under simulated occlusal loading. Results: Maximum VM stresses showed an inverse relationship with material elasticity. IPS e.max CAD exhibited the highest maximum VM stress (45.571 MPa), whereas the resin nano-ceramic showed the lowest (25.419 MPa). Despite higher stress concentrations in high-modulus ceramics, VM values for all materials remained well below their FS limits. Conclusions: All materials demonstrated adequate mechanical stability under physiological loading. Lithium disilicate showed a comparatively larger margin between stress levels and flexural strength, while lower-modulus materials tended to promote greater stress transfer to supporting structures.

## 1. Introduction

The restoration of extensive structural loss in posterior teeth continues to represent a major clinical and biomechanical challenge in restorative dentistry. Posterior dentition is routinely exposed to substantial functional and parafunctional loads, ranging from 10 N to 430 N during mastication [1], typically reaching 400–800 N in normal molar bite force [2], and in some individuals surpassing 900 N due to bruxism [3,4,5]. These considerable forces generate complex stress patterns within both the remaining tooth structure and the restorative material, making the accurate assessment of stress distribution essential for ensuring long-term restoration survival. Consequently, clinicians must carefully balance the removal of decayed or weakened tissue with the preservation of sound dental structures while selecting restorative approaches that can withstand these demanding biomechanical conditions [6]. Within this context, indirect restorations such as inlays provide a conservative yet durable alternative to full-coverage crowns [7], enabling superior preservation of natural tooth structure and contributing to improved mechanical performance [1].

Building upon these biomechanical considerations, recent advancements in computer-aided design and manufacturing (CAD/CAM) technologies have substantially transformed restorative dentistry by introducing materials with improved mechanical reliability and high dimensional precision [8,9]. This technology has introduced a great variety of restorative options, including traditional ceramics, hybrid ceramics, and resin-based materials; each characterized by distinct structural compositions and elastic behaviors [10]. Lithium disilicate glass-ceramic (e.g., IPS e.max CAD, IVOCLAR, Schaan, Liechtenstein) is highly valued for its superior esthetics, outstanding strength, [11] and balanced stress distribution under moderate loads [10]. Alternatively, Polymer-Infiltrated Ceramic Network (PICN) materials (e.g., VITA Enamic, Bad Säckingen, Germany), classified as hybrid ceramics, offer a network structure (e.g., 86% ceramic and 14% polymer) that provides a dynamic elasticity often resulting in a superior distribution of stress and a buffering effect against high forces [12]. The fundamental mechanical properties of these materials, notably their elastic modulus, significantly dictate how stress is handled: high elastic modulus materials tend to absorb and concentrate stress within the restoration itself, whereas lower elastic modulus materials, such as many resin-based systems (e.g., resin nanoceramics), tend to transfer more stress to the surrounding dental tissues and adhesive interfaces [13,14,15]. Evaluating the biomechanical response of these diverse materials is therefore essential for clinical material selection.

To rigorously evaluate the mechanical behavior of these diverse restorative materials within the complex anatomy of posterior teeth, Finite Element Analysis (FEA) has emerged as an indispensable computational method in contemporary dental research. FEA enables detailed quantification of stress and strain distributions in tooth–restoration assemblies composed of multiple materials with distinct elastic properties, under controlled loading conditions that approximate clinical function [16]. Accurate simulation relies heavily on the use of precise input parameters, particularly the Modulus of Elasticity and Poisson’s Ratio, which define each material’s elastic response [17]. For failure prediction, the von Mises equivalent stress criterion is widely employed to estimate the likelihood and localization of potential fracture within the restoration [18]. To ensure the validity of these simulations, experimental techniques such as nanoindentation, used to determine localized nanomechanical properties [19], and 3-point bending tests, used to measure flexural strength [20], are essential for generating reliable input data. Incorporating experimentally measured material properties into finite element models enhances the biomechanical relevance of numerical simulations and enables a more realistic, material-specific interpretation of stress behavior [6].

Despite extensive research utilizing FEA to evaluate stress distribution in different restoration designs and materials (including ceramics, hybrid ceramics, and composite resins), conflicting results and controversies still exist regarding the optimal choice. Crucially, most studies focus either on laboratory testing of flexural strength or numerical simulation of stress, often failing to directly associate the stress values calculated in the complex clinical anatomy (FEA output) with the inherent material strength limits (experimental flexural strength) across a broad spectrum of modern CAD/CAM material classes. Therefore, the purpose of this study is to perform a comparative biomechanical assessment of inlays fabricated from five distinct commercial CAD/CAM materials including a feldspathic ceramic (Cerec Blocs), a leucite-reinforced ceramic (IPS Empress CAD, IVOCLAR, Schaan, Liechtenstein), a resin nano ceramic (3M ESPE Lava Ultimate, Schaan, Liechtenstein), a polymer infiltrated ceramic network (VITA Enamic, Bad Säckingen, Germany), and a lithium disilicate ceramic (IPS e.max CAD, IVOCLAR, Schaan, Liechtenstein) to identify the highest stress areas under loading using the Finite Element Method. To ensure accurate input data for the finite element analysis, the elastic modulus (three-point bending) and Poisson’s ratio (nanoindentation) of each material were experimentally determined in accordance with relevant standards and incorporated into the numerical models. Within this framework, the study provides a comparative and mechanistic evaluation of how material-specific mechanical properties influence stress distributions in CAD/CAM inlay restorations. The null hypothesis was that CAD/CAM material type does not influence elastic modulus, Poisson’s ratio, flexural strength, or stress distribution patterns within inlay restorations.

## 2. Materials and Methods

The CAD/CAM materials used in this study and their chemical composition are listed in Table 1.

### 2.1. Determination of Material Properties for Finite Element Analysis

For finite element analysis (FEA), it is necessary to introduce certain mechanical properties of the materials used into the computer software. These properties are the modulus of elasticity (Young’s Modulus) and Poisson’s ratio.

**Figure 1:**
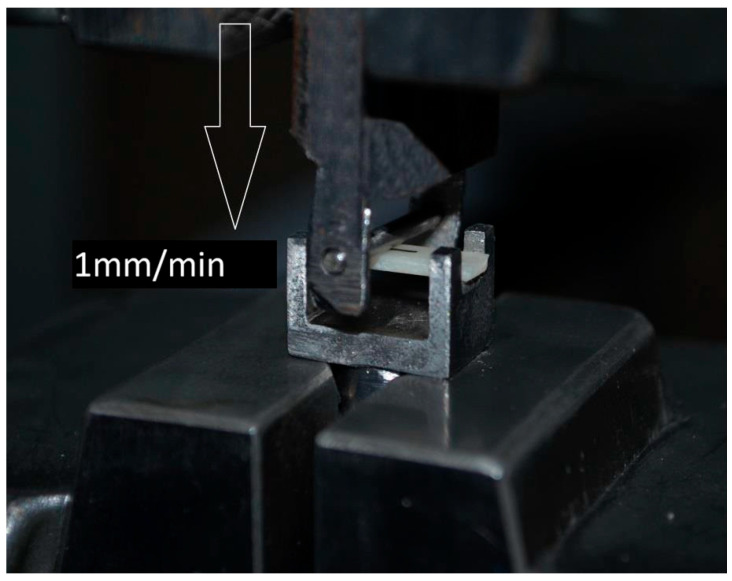
Three-point bending test setup for CAD/CAM material samples using a 2 mm diameter steel indenter.

- Modulus of Elasticity Calculation: A three-point bending test was performed to calculate the modulus of elasticity [21,22]. For this purpose, five specimens in the shape of a rectangular prism with dimensions of 15 × 4 × 1.2 mm were obtained from the ceramic blocks in each material.
- Specimen Preparation: These specimens were cut from the blocks using a slow-speed diamond saw (Isomet 1000, Buehler, Lake Bluff, IL, USA).
- Testing Procedure: The resulting specimens were placed into a custom-designed metal fixture with a width of 12 mm. The specimens were then tested until fracture by a steel cylinder with a cross-sectional area of 2 mm, which was attached to a Shimadzu Universal Testing Machine, advancing at a crosshead speed of 1 mm/min (Figure 1).

#### 2.1.1. Calculation of Modulus of Elasticity

**Figure 2:**
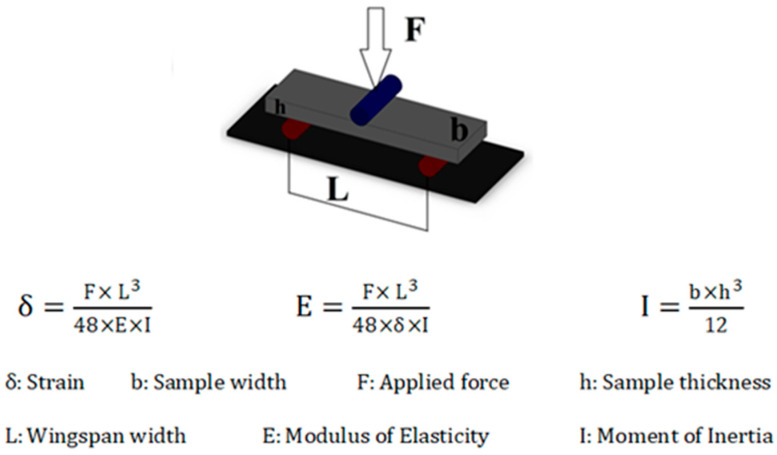
Three-point bending test scheme and flexural strength calculation formula.

The modulus of elasticity (E) values were calculated using the resulting load-deflection graphs according to the formula given in Figure 2.

#### 2.1.2. Poisson’s Ratio and Flexural Strength Determination

**Figure 3:**
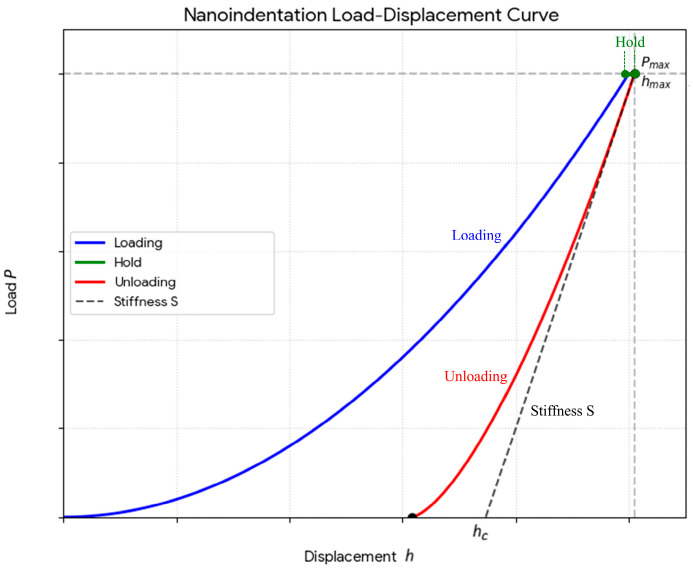
Load–displacement curve used for the calculation of Poisson’s ratio.

Poisson’s Ratio Determination: The values for the Poisson’s ratio were obtained using a nano-indentation system (IBIS nano-indentation system) [23]. Nanoindentation testing was performed on mirror-polished specimens prepared using standard metallographic procedures to minimize the influence of surface roughness on the measurements. All indentations were carried out using a Berkovich diamond indenter. A maximum load of 200 mN was applied for all materials, and the resulting load–displacement curves, illustrating the corresponding indentation depths, are presented in Figure 3. For each specimen, ten indentations were performed, with a minimum spacing of 30 µm between adjacent indents to prevent interaction effects. The load–displacement data were analyzed using the Oliver–Pharr method to determine the reduced elastic modulus, from which Poisson’s ratio was subsequently calculated using standard analytical relationships. The Poisson’s ratios were calculated based on the load-displacement graphs (Figure 3) generated during the nano-hardness measurements of the materials, utilizing the Formula (1) given below:

(1) $$E_{r}=\frac{1}{2}\times \frac{\sqrt{\pi }}{\sqrt{A}}\times S\;\;A=g\times {h_{c}}^{2}\;\;\;\;\;h_{c}=h_{max}-\varepsilon \times \frac{P_{max}}{S}\frac{1}{E_{r}}=\frac{1-{\upsilon_{i}}^{2}}{E_{i}}+\frac{1-\upsilon^{2}}{E}$$

E_r_: reduced modulus

h_max_: maximum depth

A: contact area

h_c_: contact depth

g = 24.5 (Berkovich indenter)

ε = 0.75

P_max_: Maximum force

E_i_: Elasticity modulus of indenter

υ_i_: Poisson ratio of indenter

υ: Poisson ratio of the tested material

E: Elastic modulus of the tested material

S: Slope of the unloading curve

Flexural Strength Calculation: Furthermore, in order to properly evaluate the data obtained from the finite element stress analysis, the flexural strength values of the materials are required. The mean flexural strength values of the tested materials were calculated based on the data obtained from the three-point bending test (2):

(2) σ=M×h2I σ=F2×L2×h2b×h312 σ=3×F×L2×b×h2

σ:Flexural strength

M:Momentum

h:Thickness of the sample

b:Width of the sample

F:Applied force

L:Wingspan length

### 2.2. Creation of Three-Dimensional CAD Models

**Figure 4:**
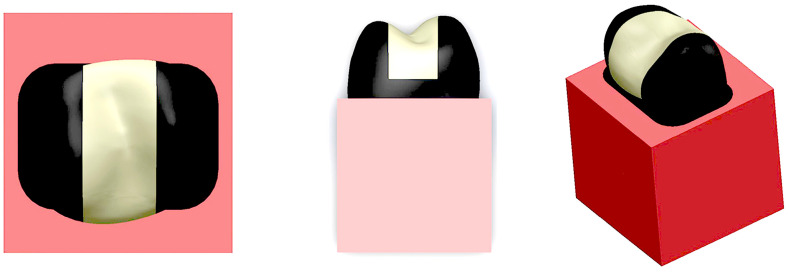
SolidWorks-based three-dimensional modeling of the inlay cavity, CAD/CAM restoration, and adjacent anatomical structures used for finite element analysis.

For the creation of the model used in this study, the following steps were undertaken:

- Imaging Data Acquisition: Images of a maxillary second premolar tooth, corresponding to the dimensions of the tooth used in our study, were obtained using the computed tomography (CT) device at the Atatürk University Faculty of Dentistry.
- 3D Model Generation: These images were transferred to the MIMICS software to generate the initial three-dimensional model.
- CAD Model Refinement and Export: The resulting model was then transferred to the SolidWorks 2014 software, where the inlay cavity, the restoration, and the surrounding structures were designed (Figure 4). These structures were finally exported with the. x_t file extension for subsequent transfer to the ANSYS Workbench 14.0 software for finite element analysis.

### 2.3. Application of Finite Element Analysis (FEA)

**Figure 5:**
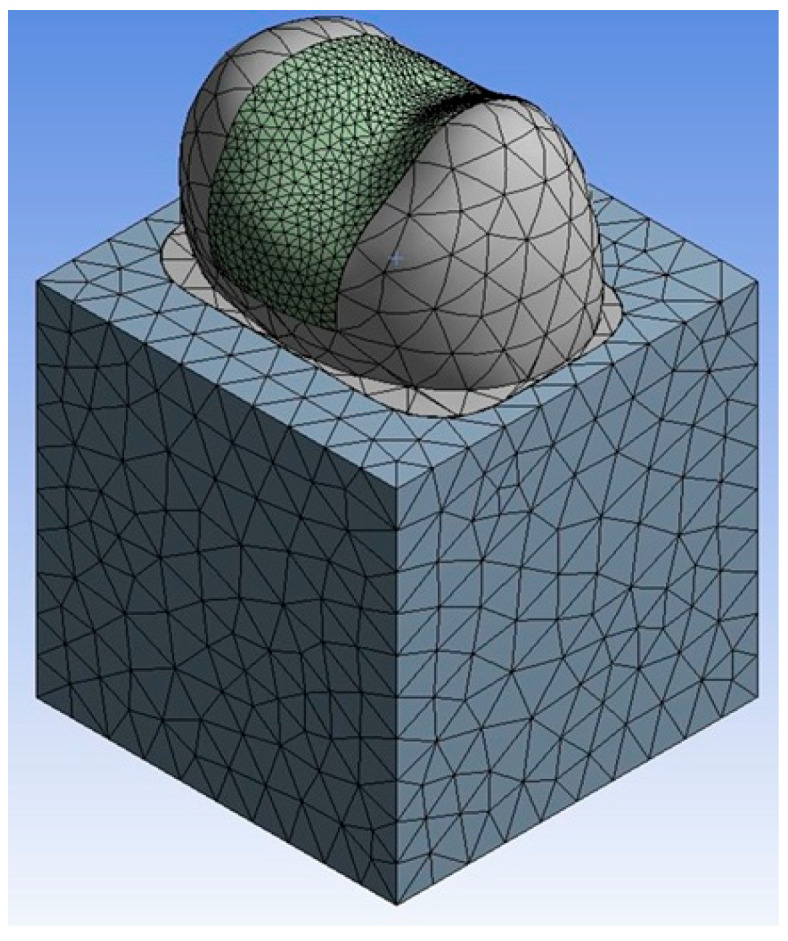
Mesh structure of 3D finite element analysis model.

**Figure 6:**
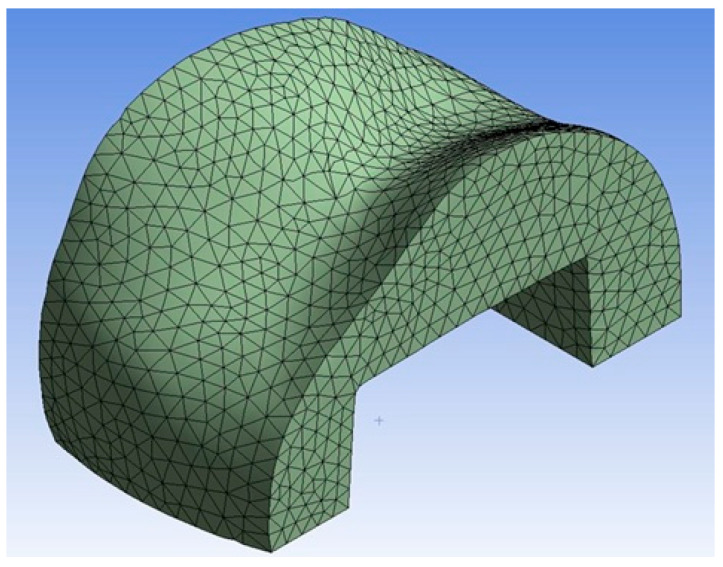
Mesh structure of the CAD/CAM inlay imported into the finite element analysis software.

This stage aimed to determine the stresses generated within the inlay restorations for each group. The finite element analyses were performed using the ANSYS Workbench software. For this purpose, the generated models were imported and meshed (optimized for compatibility) within the software (Figure 5 and Figure 6).

The resulting model was characterized by the following meshing statistics:

- Number of Nodes: 49,279
- Number of Elements: 32,243
- Element Quality Value: 0.818
- Skewness Value: 0.254

**Figure 7:**
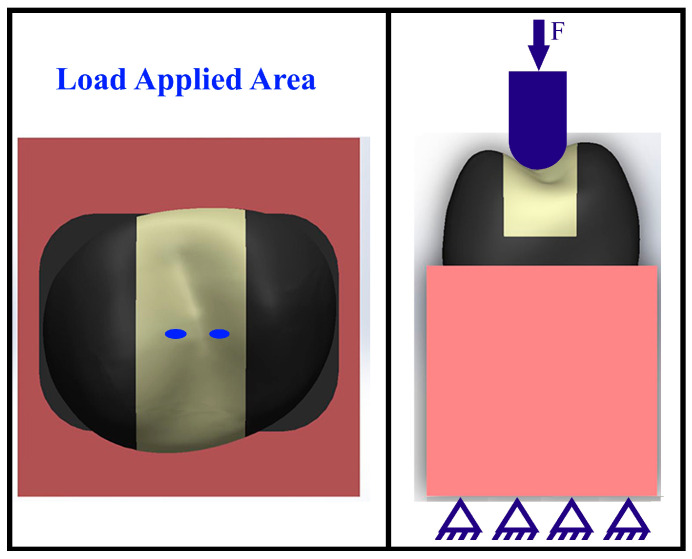
Boundary conditions and mechanical loading configuration showing the defined contact area on the CAD/CAM inlay restoration.

The finite element model incorporated clearly defined boundary conditions and contact assumptions to ensure numerical stability and reproducibility. As illustrated in Figure 7, the inferior surface of the supporting block was fully constrained in all directions to simulate rigid fixation. Occlusal loading (F) was applied to the coronal region of the tooth via a loading indenter, establishing contact with both cuspal areas of the tooth structure.

Bonded contact was assigned between the tooth and the supporting block, as well as between the restoration and the tooth structure, to represent an immobile tooth–restoration complex. In contrast, the contact between the loading indenter and the tooth surface was defined as no separation, allowing load transfer without permitting penetration or detachment during loading. These boundary conditions and contact definitions were applied consistently across all CAD/CAM inlay models. Following the definition of the contact area on the restoration, mechanical loading was performed (Figure 7). The resulting stress fields (or stress distribution) were visually evaluated.

Based on the flexural strength values obtained from the in vitro mechanical tests, a stepwise loading protocol was applied for each material in the finite element analysis. For every CAD/CAM inlay model, the applied load was incrementally increased until the calculated stress values reached or exceeded the experimentally determined flexural strength of the corresponding material. In the finite element analysis, stress thresholds associated with fracture risk were predefined for each stress criterion. Stress values exceeding the flexural strength limit were visualized using a red color scale for maximum principal stress and von Mises stress distributions, while minimum principal stress (compressive stress) values were represented using a dark blue color scale.

The load magnitude corresponding to the initial appearance of red-colored regions (for maximum principal stress and von Mises stress) or dark blue regions (for minimum principal stress) within the sectional views of the restorative material was recorded as the estimated ultimate fracture resistance of the CAD/CAM inlay. This approach enabled the establishment of a direct relationship between experimentally measured flexural strength values and numerically predicted stress concentrations.

In order to examine the stresses within the inlays under chewing forces in the oral environment, finite element analysis was performed for all CAD/CAM inlays under a 400 N load.

## 3. Results

After the three-point bending test, all specimens were observed to exhibit brittle fracture, which is typical of ceramic behavior. Upon examining the fracture data;

- The ceramic with the highest average Beam Fracture Load (BFL) was IPS e.max CAD.
- The ceramic material with the lowest average Beam Fracture Load (BFL)was IPS Empress CAD.

The amount of strain was greater for the composite-containing ceramic specimens, specifically VITA Enamic and Lava Ultimate, compared to the other specimens.

The calculated average modulus of elasticity and the material flexural strength (MFS) values are presented in Table 2.

**Figure 8:**
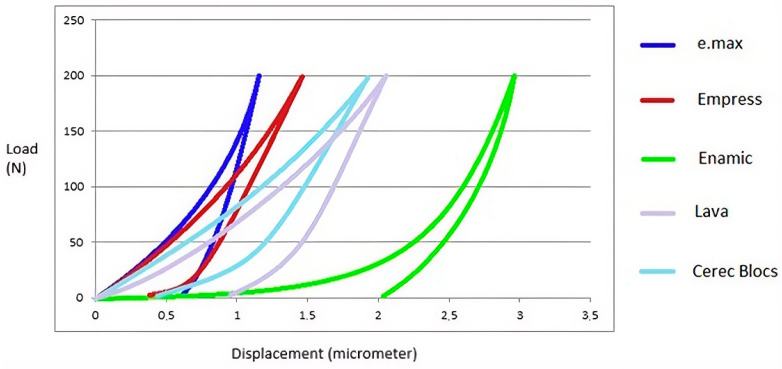
Load–displacement curves obtained from nano-indentation testing.

The average load–displacement curves obtained from nano-indentation tests, performed with 10 repetitions for each material, are presented in Figure 8. Additionally, the Poisson’s ratio values calculated from these tests are reported in Table 3.

Finite Element Analyses (FEA) were performed for five different materials based on the average fracture strength values obtained from in vitro fracture analyses.

The performed analyses investigated the first principal stresses (maximum principal stress), third principal stresses (minimum principal stress), and von Mises stress values generated in the materials, through a comprehensive examination of both the material surface and cross-sectional specimens extracted from the interior.

- First Principal Stress indicates the maximum tensile stress (tension) generated in the material.
- Third Principal Stress indicates the maximum compressive stress (compression) generated in the material.
- Von Mises stress is commonly used as a scalar parameter to represent the overall stress state and to evaluate stress distribution within materials subjected to multiaxial loading.

The results of the stress analyses conducted on the five different materials are presented in the following figures.

In accordance with the experimental setup, the inlays were subjected to compressive loading to evaluate material fracture. Consequently, the stresses generated within the inlays were predominantly compressive in nature and exhibited negative values.

**a**. **CEREC BLOCS (Load: 2100 N)**

The calculated flexural strength value for CEREC BLOCS was 156.25 MPa.

**a.1. First Principal Stress (Maximum Tensile Stress)**

**Figure 9:**
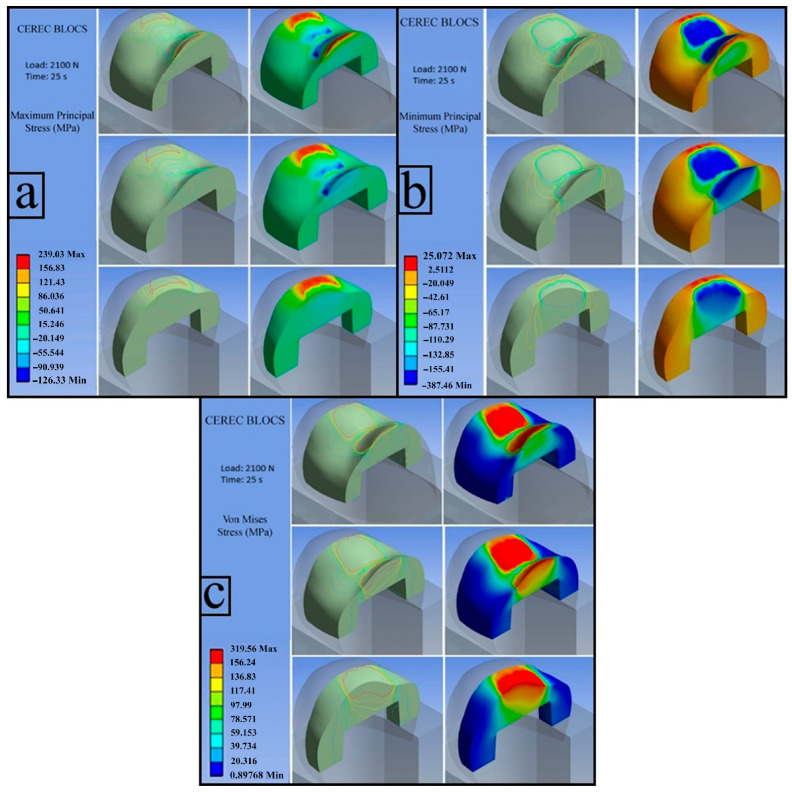
CEREC BLOCS; first principal stress (**a**), third principal stress (**b**), von Mises stress (**c**).

The distribution of the first principal stress within the inlay was analyzed, and the maximum tensile stress was found to be 239.03 MPa. Regions displayed in red exceeded the experimentally determined flexural strength and, due to their positive values, indicate the presence of tensile stresses. Compressive stresses were primarily observed in the central regions beneath the applied load, whereas tensile stresses were concentrated in the marginal areas, likely as a result of the adhesive constraint provided by the resin cement (Figure 9a).

**a.2. Third Principal Stress (Minimum Principal Stress)**

The third principal stress distribution is presented in the figure, with a maximum absolute value of −387.46 MPa, indicating dominant compressive stress. The highest compressive stresses were localized in the central region of the inlay. As the extent of regions exceeding the material’s flexural strength increased, the load-bearing capacity of the inlay cross-section diminished, ultimately leading to material fracture (Figure 9b).

**a.3. Von Mises Stress**

The red areas in the figure indicate regions where stresses exceed the MFS (156.25 MPa) of the CEREC BLOCS as determined from the three-point bending test. These regions are expected to be the initial points at which a fracture will occur. The von Mises stress distribution revealed peak stress concentrations in the central portion of the inlay. Stress values observed in the lower regions of the restoration remained below the experimentally determined flexural strength. This stress pattern suggests a progressive reduction in the effective load-bearing cross-section, followed by sudden fracture once a critical load threshold is exceeded (Figure 9c).

**b**. **IPS e.max (Load: 4000 N)**

The calculated flexural strength value for IPS e.max was 218.75 MPa.

**b.1. First Principal Stress (Maximum Tensile Stress)**

**Figure 10:**
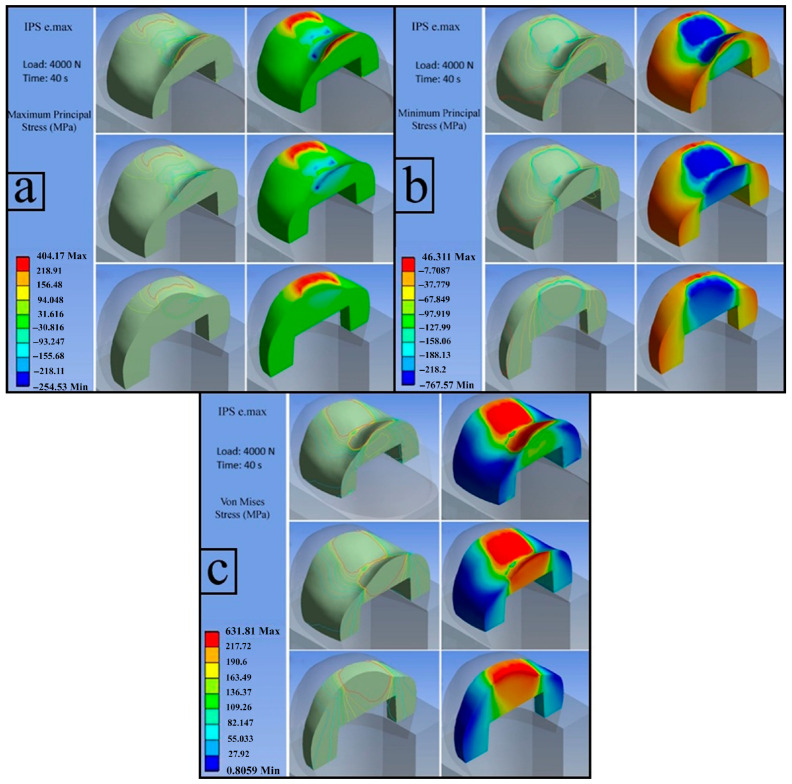
IPS e.Max; first principal stress (**a**), third principal Stress (**b**), von Mises stress (**c**).

The figure illustrates the distribution of the first principal stress, with the maximum tensile stress reaching 404.17 MPa. The red-colored regions represent stress values exceeding the experimentally determined flexural strength and indicate areas subjected to tensile loading (Figure 10a).

**b.2. Third Principal Stress (Minimum Principal Stress)**

The distribution of the third principal stress is presented in the figure, with a maximum absolute magnitude of approximately −767 MPa, confirming the presence of compressive stresses. The highest compressive stresses were concentrated in the central region of the restorative material. The development of cracks in these regions reduced the load-bearing capacity of the cross-section, ultimately resulting in structural failure (Figure 10b).

**b.3. Von Mises Stress**

The red areas in the figure indicate regions where stresses exceed the MFS (218.75 MPa) of the IPS e.max as determined from the three-point bending test. These regions are expected to be the initial points at which a fracture will occur. The von Mises stress distribution shows that stress values in the lower regions of the restoration remained below the experimentally obtained flexural strength. This stress pattern indicates a progressive reduction in the effective load-bearing cross-section, followed by sudden fracture once the material is no longer able to sustain the applied load (Figure 10c).

**c**. **IPS EMPRESS (Load: 1950 N)**

The calculated flexural strength value for IPS Empress was 115.625 MPa.

**c.1. First Principal Stress (Maximum Tensile Stress)**

**Figure 11:**
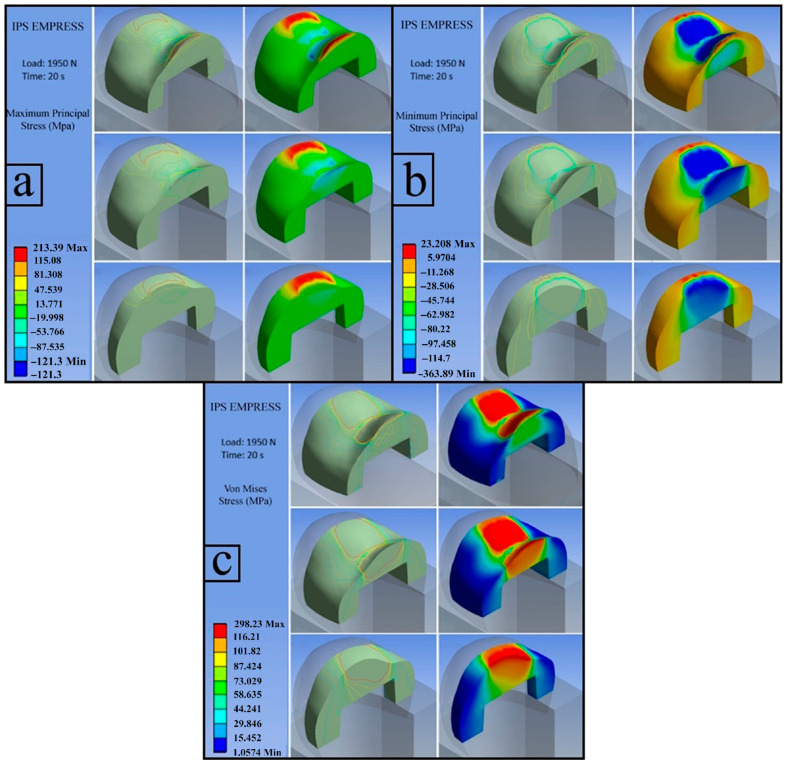
IPS EMPRESS; first principal stress (**a**), third principal stress (**b**), von Mises stress (**c**).

The figure illustrates the distribution of the first principal stress within the inlay, with a maximum tensile stress of 213.39 MPa. The red-colored regions represent stress values exceeding the experimentally determined flexural strength and, due to their positive magnitude, indicate areas subjected to tensile loading. These regions were primarily located at the upper endpoints of the restoration. The concentration of tensile stresses in these areas can be attributed to sharp geometric transitions at the upper margins, which act as stress concentrators (Figure 11a).

**c.2. Third Principal Stress (Minimum Principal Stress)**

The third principal stress distribution is presented in the figure, with a maximum absolute value of −363.89 MPa, indicating compressive stress. The highest compressive stresses were localized in the central region of the inlay. The accumulation of compressive stresses in this area led to crack initiation within the restorative material, resulting in a loss of load-bearing capacity and subsequent fracture (Figure 11b).

**c.3. Von Mises Stress**

The red areas in the figure indicate regions where stresses exceed the MFS (115.625 MPa) of the IPS EMPRESS as determined from the three-point bending test. These regions are expected to be the initial points at which a fracture will occur. Figure 11 presents the von Mises stress distribution within the inlay, highlighting the overall stress state under the applied loading conditions (Figure 11c).

**d**. **VITA ENAMIC (Load: 2450 N)**

The calculated flexural strength value for VITA Enamic was 156.25 MPa.

**d.1. First Principal Stress (Maximum Tensile Stress)**

**Figure 12:**
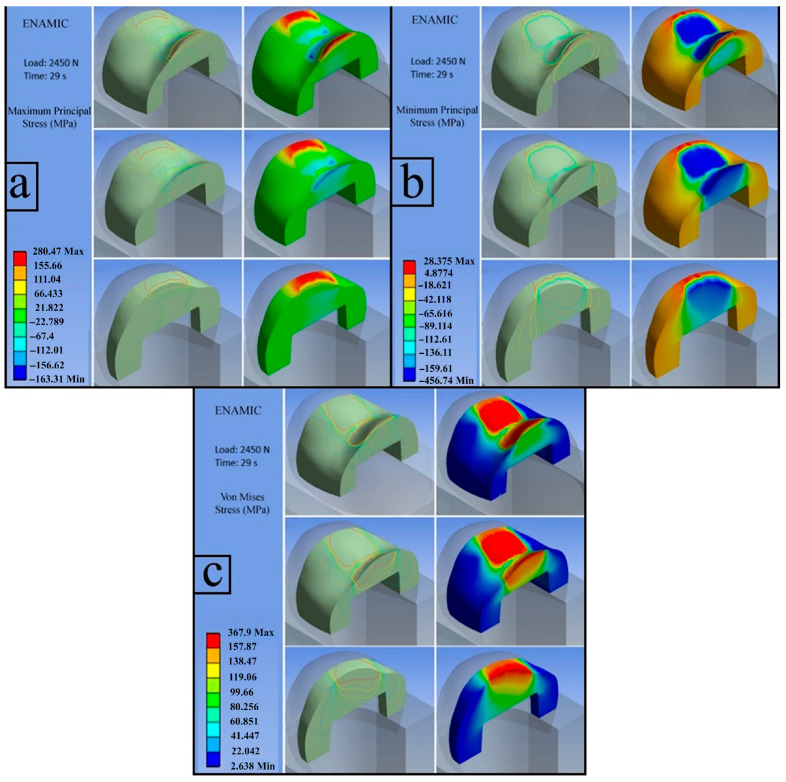
VITA ENAMIC; first principal stress (**a**), third principal stress (**b**), von Mises stress (**c**).

The figure illustrates that the red-colored regions located at the peripheral edges of the inlay correspond to stress values exceeding the experimentally determined flexural strength. As these values are positive, they indicate the presence of tensile stresses in these regions. In contrast, compressive stresses are predominantly observed in the central regions of the restoration (Figure 12a).

**d.2. Third Principal Stress (Minimum Principal Stress)**

The third principal stress distribution for the Enamic material is presented in the figure, with a maximum absolute magnitude of −456.74 MPa, confirming the presence of compressive stress. Cross-sectional analysis revealed that the highest compressive stresses were concentrated in the central portion of the inlay. The accumulation of stress in this region led to crack initiation, causing a reduction in the load-bearing capacity of the inlay and ultimately resulting in fracture (Figure 12b).

**d.3. Von Mises Stress**

The red areas in the figure indicate regions where stresses exceed the MFS (156.25 MPa) of the VITA ENAMIC as determined from the three-point bending test. These regions are expected to be the initial points at which a fracture will occur. Cross-sectional evaluation of the von Mises stress distribution demonstrates a gradual decrease in stress intensity toward the lower regions of the restoration. Although the stresses in these areas remained below the experimentally determined flexural strength, cracks initiated in the upper regions—where stress levels exceeded the material’s flexural limit—propagated readily, leading to catastrophic failure (Figure 12c).

**e**. **LAVA ULTIMATE (Load: 2400 N)**

The experimentally determined flexural strength of Lava Ultimate was 187.5 MPa. Accordingly, the stress analyses presented in the following figures focus on regions where the calculated stress values exceed this threshold.

**e.1. First Principal Stress (Maximum Tensile Stress)**

**Figure 13:**
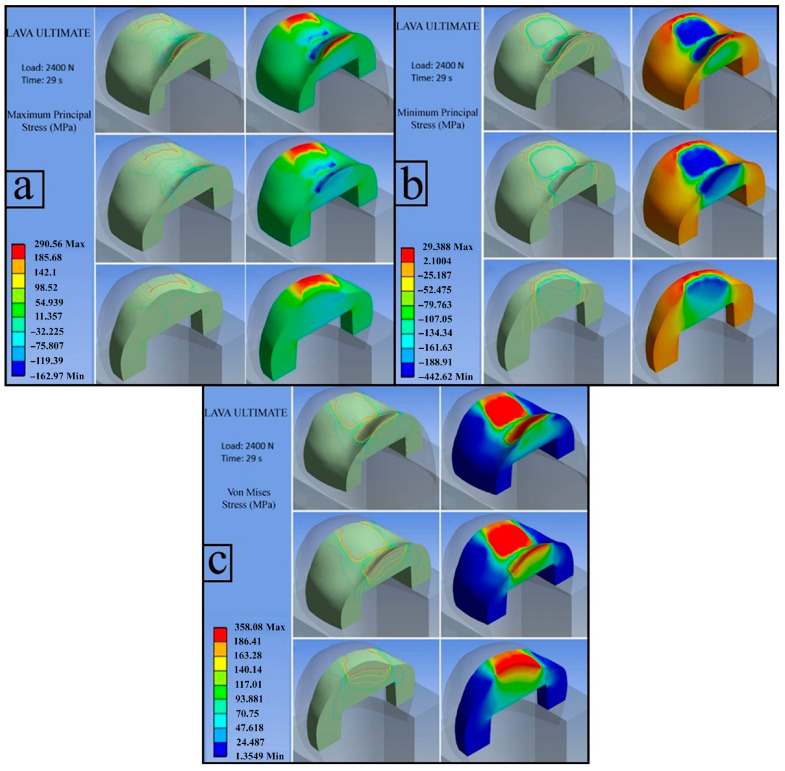
LAVA ULTIMATE; first principal stress (**a**), third principal stress (**b**), von Mises stress (**c**).

The maximum first principal stress was calculated as 290.56 MPa. Red-colored regions located at the marginal areas of the inlay represent stress values exceeding the flexural strength limit and, due to their positive magnitude, indicate tensile loading. In contrast, compressive stresses were predominantly observed in the central portions of the restoration (Figure 13a).

**e.2. Third Principal Stress (Minimum Principal Stress)**

The maximum absolute value of the third principal stress was found to be −442.62 MPa, confirming the presence of compressive stress. Stress concentrations exceeding the flexural strength in the superior regions of the inlay promoted crack initiation and subsequent crack propagation, ultimately resulting in fracture of the restoration (Figure 13b).

**e.3. von Mises Stress**

The red areas in the figure indicate regions where stresses exceed the MFS (156.25 MPa) of the LAVA ULTIMATE as determined from the three-point bending test. These regions are expected to be the initial points at which a fracture will occur. Evaluation of the von Mises stress distribution (Figure 13c) demonstrated lower stress concentrations in the inferior regions of the inlay compared with the superior aspect. Although stresses in the lower regions remained below the flexural strength limit, cracks initiated in the upper regions, where stress levels exceeded the ceramic’s flexural resistance, propagated readily, leading to catastrophic failure.

**f**. **von Mises Stress Values Under 400 N Load**

As reported in the literature, when the force applied to the tooth structure exceeds 400 N, damage to the tooth may occur. Accordingly, to compare the behavior of each material under an equal load, a stress distribution analysis was performed under a gradually applied load reaching 400 N within 10 s.

**Figure 14:**
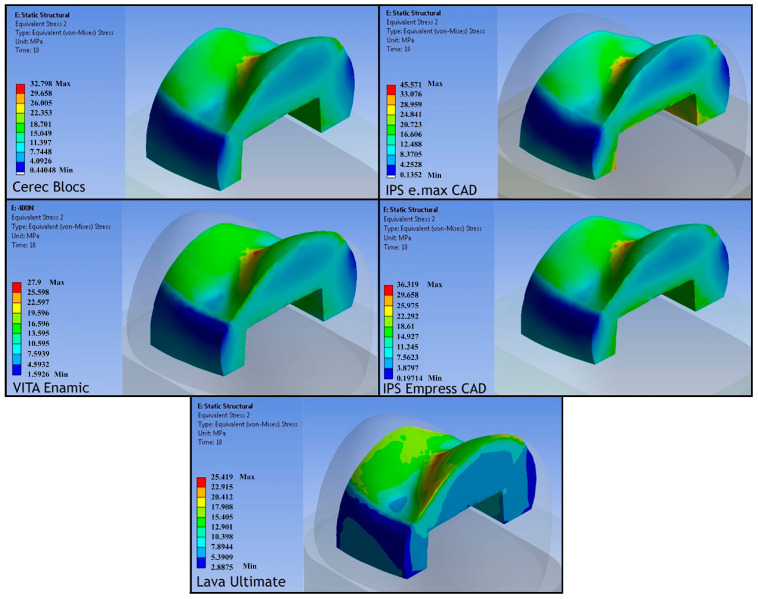
von Mises Stress Distribution within the CAD/CAM Inlay Materials.

Based on the finite element analysis performed under a 400 N occlusal load, the stress distribution within the investigated CAD/CAM inlay restorations was quantitatively evaluated using von Mises stress criteria (Figure 14). This approach enabled a comprehensive comparison of the overall stress state among materials with different microstructural compositions and mechanical properties.

The analysis demonstrated that the highest von Mises stress concentration occurred in the lithium disilicate ceramic IPS e.max, with a peak value of 45.571 MPa. This elevated stress response may be attributed to the high elastic modulus and relative brittleness of lithium disilicate ceramics, which tend to concentrate stresses rather than dissipate them under compressive loading. The leucite-reinforced ceramic IPS Empress exhibited the second-highest stress level (36.319 MPa), suggesting a comparatively improved stress distribution capacity relative to IPS e.max, yet still reflecting the inherent stiffness of glass-ceramic materials.

The feldspathic ceramic Cerec Blocs presented a moderate von Mises stress value of 32.798 MPa, indicating a more homogeneous stress distribution within the inlay. This behavior may be associated with its lower elastic modulus compared with lithium disilicate and leucite-reinforced ceramics, allowing partial stress accommodation within the material structure.

In contrast, markedly lower von Mises stress values were observed in the hybrid and resin nanoceramic CAD/CAM materials, reflecting their enhanced ability to absorb and redistribute applied loads. The VITA Enamic (polymer-infiltrated ceramic network, PICN) inlay exhibited a von Mises stress of 27.9 MPa, demonstrating a biomechanical response intermediate between ceramics and resin-nanoceramics. The lowest overall stress concentration was recorded in the 3M ESPE Lava Ultimate (resin nano-ceramic) inlay, with a value of 25.419 MPa, highlighting its superior stress-dampening capacity under occlusal loading conditions.

Overall, these findings indicate that CAD/CAM materials with lower elastic moduli and resin-containing microstructures tend to reduce stress concentration within inlay restorations, whereas stiffer ceramic materials exhibit higher von Mises stress values under identical loading conditions. This biomechanical behavior may have important implications for clinical material selection, particularly in situations where stress concentration and fracture risk are of concern.

## 4. Discussion

Finite element analysis (FEA) has become an essential numerical approach for investigating the biomechanical behavior of dental restorations, as it enables detailed, non-invasive simulation of clinical loading conditions that cannot be reproduced reliably in vivo. In the present study, a three-dimensional (3D) finite element model was generated using computerized tomography data from an extracted human tooth to maximize anatomical accuracy [24]. While both two-dimensional (2D) and 3D FEA approaches are described in the literature, the complex, irregular, and asymmetrical morphology of dental tissues limits the validity of 2D models. Consequently, 3D modeling is widely regarded as the most appropriate method for obtaining realistic stress distributions in tooth–restoration systems [25].

Previous investigations support this approach. Ona et al. [26], who compared 2D and 3D finite element models, demonstrated that 2D simulations fail to accurately represent dental anatomy and stress behavior, concluding that 3D FEA should be preferred. In agreement with these findings, all analyses in the current study were performed using a 3D model to ensure high bio-fidelity and reliable numerical outcomes.

Mesh quality represents a critical factor influencing the accuracy of finite element simulations. Although ideal skewness and element quality values are achievable in models with simple geometries, such conditions are difficult to attain in anatomically complex biological structures. Therefore, mesh optimization in the present study was achieved through a convergence analysis to determine the minimum element and node density required for stable results. The general model employed a minimum mesh size of 1.2482 × 10^−2^ mm and a maximum mesh size of 2.4963 mm, while an element size of 0.3 mm was identified as optimal for ceramic inlays. These parameters are consistent with previously published dental FEA studies and provide a balance between computational efficiency and numerical reliability.

Despite its advantages, FEA inevitably relies on simplifying assumptions, including the treatment of all materials as homogeneous, isotropic, and linearly elastic [17]. Although these assumptions do not fully capture the complex anisotropic and time-dependent behavior of dental tissues and restorative materials [27], FEA remains a powerful comparative tool for identifying stress concentration patterns and predicting potential failure regions under standardized loading conditions [19,28].

A major focus of the present investigation was to evaluate how experimentally determined mechanical properties—particularly Young’s modulus and Poisson’s ratio obtained from three-point bending and nanoindentation tests—influence stress magnitude and distribution within CAD/CAM inlay materials. The finite element analysis demonstrated that stress behavior within the restorative material is strongly governed by intrinsic stiffness and deformation capacity [18]. Materials with a high elastic modulus, such as lithium disilicate ceramics, tended to sustain higher localized stress concentrations within the restoration body, reflecting their limited ability to elastically deform under load. In contrast, materials with lower elastic modulus, including resin nanoceramics and hybrid ceramics, exhibited greater elastic deformation, resulting in lower peak von Mises stress values but a more distributed internal stress pattern [29]. These findings are consistent with previous FEA studies. Dejak and Młotkowski [30] reported that ceramic inlays, despite their higher stiffness, reached critical stress levels more rapidly than composite-based materials, a trend that parallels the present experimental results in which IPS Empress CAD exhibited the lowest fracture resistance and reached critical stress thresholds at lower loads in the numerical models. Similarly, Yamanel et al. [14] demonstrated significantly higher von Mises stress values in ceramic inlays compared with composite restorations under occlusal loading, which aligns with the present observation that all-ceramic inlays generated higher internal stresses than composite-containing ceramics under a 400 N load. Recent studies further support these findings; Vu and Doan [31] reported that composite-based inlays exhibited pronounced internal stress accumulation due to their lower stiffness and increased deformation capacity, whereas ceramic materials showed more localized but intense stress concentrations. Comparable trends were also described by Pable et al. [1] and Baldi et al. [32], who emphasized that excessive flexibility in resin nanoceramics and hybrid materials promotes internal stress buildup through increased flexural deformation, while stiffer ceramics exhibit confined stress distributions owing to their higher resistance to elastic strain. Collectively, these results indicate that internal stress development within CAD/CAM inlay materials is strongly influenced by elastic modulus and flexural behavior. An appropriate balance between material stiffness and deformation capacity appears critical for reducing stress concentration within the restoration.

The polymer-infiltrated ceramic network (PICN) material evaluated in this study (VITA Enamic) exhibited stress behavior distinct from both high-strength ceramics and resin nanoceramics. Its dual-network structure, consisting of a predominantly ceramic phase infiltrated by a polymer network, results in an elastic modulus substantially lower than that of lithium disilicate. This characteristic enables partial stress buffering and a more distributed stress pattern [9]. Previous studies have reported favorable stress redistribution with PICN materials [29]; however, some investigations have shown that lithium disilicate may generate more favorable stress conditions within the tooth structure despite higher stress concentrations within the restoration itself [18,19].

The stress patterns observed within the restorative materials in the present study are consistent with previous finite element analyses of CAD/CAM inlay restorations. Nabih et al. [33] reported similar stress distribution trends for hybrid ceramic and lithium disilicate inlays under vertical occlusal loading, with stress concentrations primarily localized within the occlusal regions of the restorative material. According to these findings, the current results demonstrated that internal stress accumulation is governed by the intrinsic mechanical properties of the material, particularly elastic modulus and flexural strength. Hybrid ceramics, characterized by lower fracture resistance, tended to exhibit higher internal stress magnitudes, whereas lithium disilicate showed a more favorable stress response under comparable loading conditions. These results suggest that increased material flexibility does not necessarily lead to stress mitigation within the restoration and may instead promote internal stress development. Overall, both the present findings and previous literature indicate that the mechanical reliability of CAD/CAM inlay restorations is primarily dictated by the material’s inherent capacity to withstand internally generated stresses under functional loading.

The results of the present study are consistent with the finite element findings reported by Del Salto Flores [34], who demonstrated that lithium disilicate inlays exhibited lower deformation and superior structural stability compared with hybrid resin restorations under high occlusal loading. In both studies, stress concentrations were primarily localized within the restorative material itself and were strongly influenced by material stiffness and fracture resistance. Lithium disilicate, characterized by higher elastic modulus and flexural strength, demonstrated the ability to sustain higher von Mises stress values with limited deformation within the modeled conditions, suggesting a distinct stress accommodation behavior compared with more compliant materials. In contrast, hybrid resin materials displayed greater deformation despite lower stress magnitudes, reflecting increased flexibility and reduced structural resistance. These findings suggest that lower stress values observed in more compliant materials do not necessarily reflect superior mechanical performance. Instead, increased strain accumulation may contribute to material instability under loading. Overall, these observations reinforce that the biomechanical performance of indirect restorations is largely governed by the intrinsic mechanical properties of the restorative material. Under the simulated loading conditions, high-strength ceramics such as lithium disilicate exhibited a more favorable balance between internal stress and deformation.

Stress evaluation in this study was performed using both von Mises stress and maximum principal stress (MPS) criteria. While von Mises stress provides a useful measure for overall stress magnitude, [1] MPS is particularly relevant for brittle materials such as ceramics, where tensile stresses govern crack initiation and fracture [17]. Therefore, stress values obtained from FEA should be interpreted alongside experimentally measured flexural strength values to enable a more informed, comparative assessment of fracture-related behavior [6].

Differences between the present findings and those of previous studies, such as Holberg et al. [35] may be attributed to variations in material properties defined within the finite element models. In the present study, Poisson’s ratios of 0.22 for IPS e.max and 0.25 for IPS Empress were used, whereas Holberg et al. employed values of 0.30 and 0.21, respectively. Such discrepancies can substantially influence stress distribution outcomes, emphasizing the importance of experimentally determined material parameters rather than reliance solely on literature values. Lithium disilicate (IPS e.max CAD), with a flexural strength typically reported around 360 MPa, demonstrated higher localized maximum principal stress values in certain regions. However, the lower flexural strength measured in the present study (218.75 MPa) primarily reflects differences in specimen geometry and testing configuration, as IPS e.max CAD blocks are supplied in limited dimensions that do not permit the preparation of larger ISO 6872–compliant specimens. Consequently, smaller specimens with a different geometry were used, which can reasonably result in lower measured flexural strength values. Despite this, due to its higher intrinsic strength, this material exhibited a comparatively greater resistance to stress concentration under the simulated loading conditions when compared to lower-strength materials such as PICN, which exhibit flexural strength values of approximately 150–160 MPa. In this context, under physiological intraoral loading conditions (400 N), von Mises stress values were used for quantitative comparison, whereas under extreme loading scenarios, maximum principal stress values were additionally evaluated to better reflect fracture-related behavior in brittle ceramic materials. For example, under an extreme simulated load of 4000 N, far exceeding masticatory forces, IPS e.max CAD exhibited stress levels approaching its reported flexural strength, whereas lower-strength materials reached comparable stress levels at lower applied loads.

When the finite element analysis (FEA) findings are interpreted together with the mechanical test results obtained in the present study, it becomes evident that the fracture behavior of CAD/CAM materials is governed not only by the magnitude of stress generated within the material but also by the combined influence of flexural strength, elastic modulus, and deformation capacity. The three-point bending test demonstrated that IPS e.max CAD exhibited higher flexural strength values within the tested sample set, which helps explain its ability to tolerate higher numerically calculated stress levels without early failure in the present simulations. In contrast, the lower flexural strength of IPS Empress CAD was consistent with its greater susceptibility to material failure at comparatively lower stress levels observed in the numerical analysis.

Composite-containing ceramics, such as VITA Enamic and Lava Ultimate, exhibited lower elastic modulus values and greater deformation behavior in both bending and nanoindentation tests, which was reflected in the FEA results as a more distributed internal stress pattern within the material. However, this increased deformability was accompanied by higher deflection values, indicating a more compliant mechanical response compared with conventional glass ceramics. In contrast, IPS e.max CAD, characterized by higher elastic modulus and flexural strength, demonstrated superior resistance to internally generated stresses, sustaining higher stress levels in the FEA simulations while exhibiting limited deformation and higher Beam Fracture Load (BFL) experimentally. The Beam Fracture Load (BFL) corresponds to the load at which the specimens prepared for the three-point bending test fractured. Based on this Beam Fracture Load (BFL), the flexural strength values of the materials were calculated. Without the determination of the Beam Fracture Load (BFL), it is not possible to derive the material strength values normalized to unit area. These findings are in agreement with Melc et al. [36], who reported that glass ceramics and hybrid CAD/CAM materials present significantly higher flexural strength than feldspathic ceramics and resin-based composites, and that materials with higher stiffness are better able to resist bending-induced stresses through reduced deformation. Similar to their observations, the polymer-infiltrated ceramic network structure of VITA Enamic in the present study was associated with increased deflection and a relative reduction in flexural performance, suggesting a greater susceptibility to internal stress accumulation under bending loads. Furthermore, the load–displacement behavior observed herein is consistent with the findings of Ramos et al. [37], who demonstrated that CAD/CAM composite blocks exhibit higher flexural strength and stiffness than direct composite resins, and limited deformation prior to failure. Likewise, materials with lower elastic modulus in the present study showed increased deflection under bending loads, whereas stiffer materials sustained higher internal stresses with reduced deformation. All tested materials exhibited brittle fracture behavior, supporting previous reports that failure in CAD/CAM restorative materials is primarily governed by intrinsic material properties rather than plastic deformation. In addition, the present findings corroborate the work of Alanazi et al. [38], who emphasized the critical role of flexural strength and elastic modulus in determining deformation behavior and failure patterns in hybrid ceramics. Collectively, these results suggest that peak stress values alone may not fully characterize the mechanical response of CAD/CAM restorative materials. Elastic modulus and flexural strength have important role in determining how stresses are distributed within the material under simulated loading conditions. Accordingly, the combined consideration of experimentally measured mechanical properties and FEA-derived stress distributions allows for a more nuanced, comparative interpretation of material behavior within the numerical framework of the present study, without implying direct prediction of clinical safety margins.

Several limitations of this study should be acknowledged. First, the finite element analysis was based on static loading conditions and did not incorporate fatigue behavior, flaw sensitivity, subcritical crack growth, or aging-related degradation, all of which are known to play a critical role in the clinical fracture of brittle ceramic materials. It should be emphasized that the flexural strength results obtained in this study were derived from a limited sample size and were not subjected to Weibull or probabilistic reliability analysis. Consequently, the stress-based findings should be interpreted as comparative indicators of mechanical response under controlled loading scenarios that support the numerical stress analysis, rather than as predictors of long-term clinical performance or failure probability. Second, the applied loading conditions, although selected to represent both physiological and extreme forces, cannot fully replicate the dynamic and multidirectional nature of occlusal loading encountered clinically. Furthermore, biological factors such as periodontal ligament behavior and individual variations in tooth anatomy were not incorporated into the model.

Future studies should aim to incorporate fatigue analysis, thermomechanical loading, and more complex material models to better approximate clinical conditions. Including cohesive zone modeling at adhesive interfaces and patient-specific geometries may further enhance predictive accuracy. Additionally, combining FEA with long-term in vitro fatigue testing and clinical outcome data would provide a more comprehensive understanding of the biomechanical performance and durability of CAD/CAM inlay materials.

In addition, future investigations should include larger sample sizes and, where feasible, standardized flexural testing protocols to reduce uncertainty associated with specimen geometry and testing configuration. The adoption of fracture-mechanics–based failure criteria, as well as probabilistic approaches that account for material flaw size and distribution, would allow a more realistic assessment of crack initiation and propagation in brittle CAD/CAM materials. Moreover, experimental validation of finite element predictions using restored teeth subjected to load-to-failure and cyclic fatigue testing would provide a stronger experimental–numerical linkage and improve the translational relevance of FEA-based stress analyses.

## 5. Conclusions

Overall, the present study demonstrates that the biomechanical behavior of CAD/CAM inlay materials is governed by the interplay between intrinsic mechanical properties and stress distribution characteristics under simulated loading conditions. High-strength glass ceramics, such as IPS e.max CAD and IPS Empress CAD, exhibited higher internal stress concentrations, as reflected by elevated von Mises and maximum principal stress values. However, these stress levels must be interpreted in relation to the experimentally measured flexural strength and elastic modulus, rather than being considered independent indicators of failure risk.

In contrast, hybrid and resin-based materials, including VITA Enamic and Lava Ultimate, demonstrated lower peak stress values and a more distributed stress pattern, which reflects their greater deformation capacity rather than superior mechanical resistance. Accordingly, lower stress magnitudes within these materials should not be interpreted as an inherent mechanical advantage without consideration of their reduced strength limits and increased strain accumulation.

By integrating quantitative finite element–derived stress metrics with experimentally measured mechanical properties, this study provides a comparative assessment of stress behavior among different CAD/CAM inlay materials under standardized loading conditions. However, the findings should be interpreted within the limitations of static loading, simplified material assumptions, and the absence of fatigue, aging, and flaw-sensitive fracture mechanisms. Consequently, the results do not aim to predict clinical failure but rather to elucidate relative biomechanical trends that may inform material selection and guide future experimental and numerical investigations.

## Figures and Tables

**Table 1:** CAD/CAM materials used in this study and their compositions.

Material	Brand	Type	Composition (% wt)
Cerec Blocs,	SIRONA, Charlotte, NC, USA	Feldspathic	SiO_2_ 56–64, Al_2_O_3_ 20–23, Na_2_O 6–9, K_2_O 6–8, CaO 0.3–0.6, TiO_2_ 0.0–0.1
IPS Empress CAD	IVOCLAR, Schaan, Liechtenstein	Leucite-reinforced glass ceramic	SiO_2_ 60–65, Al_2_O_3_ 16–20, K_2_O 10–14, Na_2_O 3.5–6.5, Other Oxides 0.5–7.0, Pigments 0.2–1.0
IPS e.max CAD	IVOCLAR, Schaan, Liechtenstein	Lithium disilicate glass ceramic	SiO_2_ 57–80, Li_2_O 11–19, K_2_O 0–13, P_2_O_5_ 0–11 ZrO_2_ 0–8, ZnO 0–8, Al_2_O_3_ 0–5, MgO 0–5
Lava Ultimate	3M ESPE, St. Paul, MN, USA	Resin nano ceramic	Ceramic (80%) Resin (20%)
Enamic	VITA, Bad Säckingen, Germany	Hybrid Ceramic	Ceramic (86%): SiO_2_ 58–63, Al_2_O_3_ 20–23, Na_2_O 6–11, K_2_O 4–6, B_2_O_3_ 0.5–2, CaO < 1, TiO_2_ < 1 Polymer (14%): PMMA

**Table 2:** The mean Beam Fracture Load (BFL), deformation, flexural strength, and elastic modulus values of CAD/CAM materials were obtained from the three-point bending test.

CAD/CAM Material	Beam Fracture Load (N)	Beam Fracture Strain (mm)	Material Flexural Strength (MPa)	Modulus of Elasticity (GPa)
e.max	70.15 ± 3	0.039	218.75 ± 16	101 ± 5
Empress	30.83 ± 1	0.033	115.62 ± 9	60 ± 2
Enamic	50.20 ± 2	0.084	156.25 ± 11	36 ± 2
Lava	60 ± 2	0.219	187.50 ± 13	17 ± 1
Cerec Blocs	50.20 ± 2	0.063	156.25 ± 10	49 ± 3

**Table 3:** The Poisson ratios of CAD/CAM materials calculated from nano-indentation tests.

CAD/CAM Materials	e.max	Empress	Enamic	Lava	Cerec Blocs
**Poisson’s Ratio (ν)**	0.22	0.25	0.33	0.36	0.23

## Data Availability

The original contributions presented in the study are included in the article, further inquiries can be directed to the corresponding author.

## Abbreviations

The following abbreviations are used in this manuscript:

FEA	Finite Element Analysis
CAD/CAM	Computer-Aided Design/Computer-Aided Manufacturing
FS	Flexural Strength
VM	Von Mises
E	Modulus of Elasticity
CT	Computed Tomography
PICN	Polymer-Infiltrated Ceramic Network
